# Event and Comment

**Published:** 1917-08

**Authors:** 


### DENTAL REGISTER

Vol. LXXI AUGUST, 1917 No. 8

### EVENT AND COMMENT

THE IVORY CROSS. This is the title of a new society just formed in London to take over the care of funds raised by voluntary subscription, for improving the condition of English soldiers and their dependents so far as their teeth are concerned. The Government is doing much for the soldiers at the front or before they get to the front; but there is a lot of dentistry needed for the returned soldiers and sailors, whose dental conditions are badly disorganized by the hardships of their army experiences. The meeting called at the Lord Mayor's House in London, was very enthusiastic in so far as the speeches went, and a sum of over $5000 was subscribed to start the work. This incident will be worth while considering by our people, as no doubt when our soldiers begin to return from their European service, they will need much reconstructive dental service. It is very probable that our men go into the war better prepared dentally, and it is hoped we may send along enough dentists to keep them in fairly good condition. In spite of this advantage, we shall undoubtedly be called upon for a considerable greater service than under ordinary conditions. It will, therefore, be well for us to see to it that we are prepared to serve these men adequately when they return home, as well as to send competent dentists with them to the front.

LEGISLATION FOR THE ARMY AND NAVY DENTAL CORPS. We are printing in this issue an appeal from Dr. H. C. Brown, chairman of our National Legislation Committee, for help to secure the legislation we have so long sought, which will put our army dentists in the same rank that our medical brothers now have. It seems strange to us that legislation of this character should be held up so long. There can be no good argument advanced against it, and there is an abundance of testimony from disinterested sources to justify the profession's claim for suitable recognition for the men who are making a sacrifice of opportunity in order that they may show a proper patriotic attitude in these times. But legislative bodies are timid organizations, at least they are proverbially conservative, and need to be assured by their constituents before taking any new steps of a radical character. If it is not too late when you read this, drop a line to your congressman and ask him to do what he can to promote this legislation. It is a proper measure, and your interest may be of great value in clearing up the mind of your congressman. Do it any way that seems best to yourself.

EDITOR DENTAL REGISTER. *Shall members of the profession enlist as dental surgeons, or be drafted into the ranks as fighting men?*

The great importance of the dental profession in the present world war has never had its counterpart in any previous war. The knowledge of results attained through dental service has received so large a recognition that today we find ourselves in the very front of governmental activities, having to do with the preservation of the health of all men under arms, as well as taking no small part in the restoration of wounded. Hardly more than fifteen years have passed since dental service received in part its due recognition by our government. Surgeon Col. Owen's interesting experience related in one of the late journals, concerning what is probably the first dental service rendered our armies, takes us back only to the American intervention in the Philippines. So today we are called upon to give our best, do our best, not considering such to be a sacrifice, but a privilege and an opportunity to serve in a large way towards the preservation of our country's national honor, its integrity and its world teaching democracy.

Two meetings have been held in Washington since the declaration of war, participated in by dental educators, State Examining Board secretaries and various officers of our larger dental societies. The first on May 12 and the next on July 18.

The activities of Dr. E. C. Kirk and latterly Dr. Wm. H. G. Logan as chairmen respectively of the Dental Committee of the medical section of the Council of National Defense, show to what an extent dental service is expected as we take our place in the tremendous warfare abroad.

Just now the most serious situation which confronts us is the fact that a large number of the members of our profession must enlist in the dental section of the Officers' Reserve Corps. Initiative as to action must be manifest, because the work is very new, and the unexpected will often happen. Our young men should recognize that there is a greater reason for war service than the probabilities of the draft with its uncertain assignment to the proper place when drafted. We are confronted during the formation of this new third, or national army, with the fact that neither dental nor medical practitioners, teachers nor students are exempt from the draft. No doubt, in the organization of the first unit, the special attainments of the men called to form it will receive careful analysis and probably due consideration. We can rest assured that the government will not ignore entirely the great need of dental and medical service at home as well as abroad. The very great loss of the dental and medical profession to Great Britain and other countries early in the war is well known to all, yet we should not doubt the justness of the government's insistence that selective exemption in its entirety is not the right stand. Experiences of the other countries, no doubt, will lead to proper recognition of the desire to maintain, without fatal sacrifice, the integrity of the medical and dental educational situation.—Dr. H. M. Semans, D.D.S., Dean, Ohio State University, Columbus, O.

---

### IS PYORRHEA ALVEOLARIS CURABLE?

The editor of *Around the Table* in the July *Dental Items of Interest* prints replies from several specialists, some of which we reprint below:

"Have our eminent periodontists had sufficient experience in the treatment of the mouth to be able to assure patients who place themselves completely in their control, as to advice and prophylactic care, that they will never become victims of pyorrhea? Second; in cases where pyorrhea already exists, what promise of cure can be made?"

Dr. Arthur H. Merritt, of New York, says: "In reply to question one, I would say that it is my belief and experience that patients with no pyorrheal lesions, who place themselves in the care of the competent periodontist, and who will follow his instructions, can be absolutely assured of never becoming the victim of pyorrhea. There is probably no disease to which the mouth is subject, so easily prevented. If dentists would only realize that every case of gingival irritation is a potential pyorrhea, and would take steps to remove the causes of such irritation, and at the same time would instruct their patients in the proper care of their mouths, most of those cases which now find their way into the hands of the periodontist would never have developed. There are of course other factors, such as occlusion, nutritional disturbances of the investing tissues of the teeth, possible systemic complications, etc., but intelligent care would suffice to prevent their ever becoming sufficiently potent to actually bring about pyorrhea.

Under such conditions one need have no hesitation whatever in assuring patients 'that they will never become the victim of pyorrhea.'

"In cases where pyorrhea already exists, the promise of cure can, within certain limits, be made with equal assurance. That there are incurable cases, where the disease has progressed to the point where there is no longer any bone attachment about certain teeth, or where so many have been lost as to impose such strain upon those remaining, as to make prognosis doubtful must be obvious. But where these conditions do not exist, and the teeth are vital, pyorrhea can be cured. This can not be made too emphatic. By this is meant restoration to health, obliteration of pockets and regeneration of bone with attachment. Anything less than this can not be regarded as a cure. Such results can not be obtained in cases where the teeth are pulpless or in traumatic occlusion, or where imperfect root surgery has been practiced. The explanation for so much skepticism regarding the cure of pyorrhea is to be found, not in the incurable nature of the disease, but in the unskilful way in which it has been treated. Poor root surgery, even when supplemented by vaccines and all the nostrums so loudly advocated, never has and never will cure a single case of pyorrhea. But in those cases in which good root surgery has been practiced, proper attention given to occlusion, and the patient instructed in intelligent home care, cure follows as a natural course of events."

Dr. Austin F. James, of Chicago, writes as follows: "My answer to your first question is unqualifiedly, yes. To the second; a cure can be accomplished in all cases where there is enough bone left to support the tooth and the mechanical strain is not unduly heavy. Failure in the first case would prove the periodontist to be either a poor teacher or a poor operator."

Dr. Paul R. Stillman, of New York, expresses his views thus: "Prophylactic care of the mouth, as it is now understood, includes not alone mouth sanitation as practiced by the dental hygienist, but also the elimination of each of the several contributing factors which complicate the treatment of almost every case of pyorrhea. Chief among these is an acquired functional mal-relation as found in the occlusion, and which includes a trauma of the pericementum. This mal-relation may be induced by attrition, resulting in a loss of occlusal enamel tissue and a coincident deformity of the normal occlusal contour; by congenital mal-occlusion; or by drifting of the teeth themselves. Instances of the latter are found in mouths where the loss of teeth by extraction or by incorrect attempts to restore the occlusal surfaces by crowns, bridges, inlays or fillings have distorted the normal anatomical co-ordination of the occlusion. It is reasonable to believe that a patient in whose mouth the correct occlusal relations are preserved, receiving and practicing proper prophylaxis will never become a victim of pyorrhea.''

I reply to your second question as follows: ''One does not promise cures. Basing prognosis upon my own experiences, the percentage of cases successfully treated and cured is very high. By cure is meant a return of the tissues to normal health. Individual teeth are very frequently encountered with dental periclasia so far advanced that the usual treatment is contraindicated. These teeth are extracted. I am amazed that any 'prominent dentist' would seriously ask these questions.''

Dr. A. L. Hamm, of Denver: ''I would say it is my firm belief that in cases where no pyorrhea exists, provided the occlusion is normal, and there are no undermining constitutional disturbances such as anaemia, malaria, syphilis, metal poisoning and other conditions of like nature; and where the patient co-operates in full harmony with the periodontist, pyorrhea will never develop.''

In reply to your second question, ''In cases where pyorrhea already exists, what promise of cure can be made?'' ''I might say that I never use the word 'cure,'

but prefer the terms, 'control' and 'arrest.' In the great majority of cases where treatment is indicated, that is, where the prognosis is favorable, an arrest or control of the condition can be secured.

"But this result can be maintained only by full co-operation of the patients with the periodontist, which constitutes prophylactic treatments from time to time, and eternal vigilance on the part of the patients in their daily care of the oral cavity. Pyorrhea is not a specific germ disease; therefore an immunity can not be created, and the causative factors of the condition can not be permanently eliminated.

"I firmly believe that the periodontists have developed their technic to such a degree, that, in the great majority of cases, beautiful results are obtained, and teeth doomed to inevitable exfoliation are saved to the patient in a normal, efficient condition for time indefinite. I trust that I have made myself clear in differentiating between a permanent cure and a permanent control or arrest of pyorrhea."

Dr. Jules J. Sarrazin, of New Orleans, has the following views: "If we more clearly realized that Riggs' disease, like any other, results from infection, we would better appreciate the importance of minimizing micro-organic life in the mouth. According to the resistance of tissues, it requires more or less of it to cause disease. As we have no means other than results to measure tissue resistance by, the only safeguard is thorough, effective home prophylaxis, backed by as much chair prophylaxis as the patient's shortcomings may necessitate. That combination, thoroughly applied, will reliably prevent pyorrhea, even though alveolar atrophy may in time destroy supporting tissue until a proportion of the length of roots become exposed, too much for the imbedded portion thereof to withstand morsal stress. Such a condition results from dystrophia, and is amenable only to timely systemic measures, helped by physiologic gum massage. With the combination of accurate curetage of roots and sockets, effective and not destructive germicidal treatment, systemic measures against cell poisoning and destruction, and prophylaxis at home and at the chair, patients may be assured that recurrence of the disease need not be feared as long as they properly maintain their home prophylaxis. Otherwise, reinfection occurs. 'Riggs' disease,' because it depicts no stage of the malady, may be taken to mean any phase of it: congestion, inflammation or suppuration. It may even include etiologic infection, and applies to dystrophia and atrophy. 'Pyorrhea' means only that stage when pus exudes."

Dr. F. H. Skinner, of Chicago, phrases it thus: "I believe one is safe in giving a favorable pyorrhea prognosis under the following conditions: Where teeth are in good alignment and occlusion, or can be made so. When not over half the process has been destroyed, as evidenced by radiographs. When the pockets have not extended between bifurcated roots. When the patient has the ability and willingness to do the necessary work at home. When prophylaxic treatments are continued after the pyorrhea has been cleared up. Where pockets extend between roots of molars or bicuspids, thorough work and persistent care may make those teeth useful for a period of years, providing occlusion which does not produce irritation can be obtained. In other words, the earlier a case is taken the better the prognosis. Ideal prophylaxis should be started before any pathological conditions have developed."

